# Cimifugin inhibits adipogenesis and TNF-α-induced insulin resistance in 3T3-L1 cells

**DOI:** 10.1515/med-2023-0855

**Published:** 2023-11-29

**Authors:** Xiang Deng, Zhenmin Liu, Siqi Han

**Affiliations:** Department of Pediatrics, Chengdu Fifth People’s Hospital, No. 33, Mashi Street, Wenjiang District, Chengdu, Sichuan, 611130, China; Department of Pediatrics, Chengdu Fifth People’s Hospital, Chengdu, Sichuan, 611130, China

**Keywords:** obesity, cimifugin, adipogenesis, insulin resistance, inflammation, MAPK pathway

## Abstract

To investigate the effects of cimifugin on adipogenesis and tumor necrosis factor (TNF-α)-induced insulin resistance (IR) and inflammation in 3T3-L1 adipocytes. 3T3-L1 adipocytes were treated with 3-isobutyl-1-methyl-xanthine, dexamethasone, and insulin or cimifugin and then Oil Red O staining and intracellular triglyceride content detection were performed to assess adipogenesis. Subsequently, after cimifugin treatment, TNF-α was used to induce IR and inflammation. The results showed that cimifugin reduced intracellular lipids accumulation of 3T3-L1 adipocytes. Cimifugin improved IR of 3T3-L1 adipocytes induced by TNF-α, as reflected in decreased adiponectin, GLUT-4, and IRS-1 mRNA and protein expression. Moreover, cimifugin reduced TNF-α-induced pro-inflammatory factors production and phospho-P65 expression, and MAPK pathway activation in the 3T3-L1 adipocytes. These findings suggested that cimifugin might be useful for the prevention and therapy of obesity-related IR and inflammation.

## 1 Introduction

Obesity has become a worldwide epidemic, which seriously affect children and adolescents’ physical and psychological development [1,2]. Obesity is characterized by an excess or abnormal distribution of fat content throughout the body [3]. Moreover, an elevated release of free fatty acids, reactive oxygen species, and pro-inflammatory factors within adipose tissue contribute to the development of insulin resistance (IR), thereby augmenting the susceptibility to various obesity-related diseases [4–6].

Cimifugin, a traditional Chinese medical herb also called Fang-feng, is a coumarin derivative obtained from the root of *Saposhnikovia divaricata*, which exhibits diverse biological properties against allergy, inflammation, and oxidative stress [7,8]. It has been reported that cimifugin inhibited inflammation and oxidative stress during psoriasis-like pathogenesis [9]. In mice with type 2 atopic dermatitis, cimifugin administration reduced epithelial cells’ allergic inflammation [10]. Cimifugin protected hepatocytes from lipotoxicity-induced death and steatosis [11]. However, few articles have reported on cimifugin’ application in the field of obesity and its lack of mechanism of action.

The elevation of tumor necrosis factor (TNF-α) in adipose tissue of individuals with obesity has been substantiated [12]. As an inflammatory factor released by adipocytes, it not only regulates cellular survival, apoptosis, and cytotoxicity, but also exhibits a strong association with the development of obesity-induced IR [13,14]. Herein, the role and possible mechanisms of cimifugin on adipogenesis and TNF-α-induced IR and inflammation were investigated in 3T3-L1 cells.

## 2 Materials and methods

### 2.1 Cell culture and differentiation

3T3-L1 preadipocytes were acquired from ATCC (Manassas, VA, USA), and cultured in Dulbecco’s modified eagle medium (DMEM) (Sigma-Aldrich, St. Louis, MO, USA) with 10% FBS and 1% penicillin/streptomycin at 37℃. To induce differentiation, 3-isobutyl-1-methyl-xanthine, dexamethasone, and insulin (MDI) induction medium was used. Preadipocytes at 2 days postconfluence were incubated with 10% FBS-DMEM supplemented with 0.5 mM 3-isobutyl-1-methyl-xanthine, 1 µM dexamethasone, and 1 µg/mL insulin (MDI medium). After 2 days, the medium was replaced with 10% FBS-DMEM containing 1 µg/mL insulin, and 10% FBS-DMEM was replenished every 2 days until mature adipocytes were obtained.

### 2.2 Cell counting kit-8 (CCK8) assay for cell viability

3T3-L1 adipocytes in 96-well plates (1 × 10^3^/well) were treated with 0–200 mg/L of cimifugin (IC0410, Solarbio, Beijing, China) for 12 h. Next, 10 µL CCK8 solution (Beyotime, Shanghai, China) was used and incubated in each well for 1 h. Then, a 450 nm absorbance measurement was performed. After that, 3T3-L1 adipocytes were subjected to pretreatment with 0, 25, 50, and 100 mg/L of cimifugin for 1 h, and then exposed to 5 ng/mL TNF-α for 24 h, and then cell viability was determined as described above.

### 2.3 Lipid quantification

To evaluate lipid accumulation, Oil Red O staining was performed. 3T3-L1 adipocytes were treated with MDI or cimifugin (25, 50, or 100 mg/L), washed, fixed with 4% formaldehyde for 1 h, and then incubated with 3 mg/mL ORO solution for 1 h. Next, the cells were washed and pictured. Further, isopropanol was utilized to elute the dye within the cells, and the lipid accumulation was quantified by detecting 520 nm absorbance.

The intracellular triglyceride (TG) content of 3T3-L1 adipocytes was determined enzymatically using a TG kit (Wako Chemicals, Richmond, VA, USA).

### 2.4 Enzyme-linked immunosorbent assay (ELISA)

The culture supernatant of 3T3-L1 adipocytes after TNF-α and cimifugin treatment was collected, and the levels of Interleukin 6 (IL-6), IL-1β, and monocyte chemotactic protein-1 (MCP-1) were assessed using ELISA kits (R&D Systems, Minneapolis, MN, USA).

### 2.5 Quantitative PCR (qPCR)

Trizol reagent (Servicebio, Wuhan, China) was employed to isolate total RNA, and a Fastquant reverse kit (TIANGEN, Beijing, China) was utilized to synthesize cDNA from total RNA. Subsequently, ABI 7500 system (Applied Biosystems, Carlsbad, CA, USA) was used to conduct qPCR using SYBR Green method. The sequences of primers is given in Table 1. Calculation of mRNA relative levels was based on the 2^−ΔΔct^ method, which normalized to β-actin.

**Table 1:** Primer sequences for qRT-PCR

Gene	Forward (5′–3′)	Reverse (5′–3′)
Adiponectin	ACTGCAGTCTGTGGTTCTGA	CATGACCGGGCAGAGCTAAT
GLUT-4	GTTCTTTCATCTTCGCCGCC	TTCCCCATCTTCGGAGCCTA
IRS-1	AGAGGACCGTCAGTAGCTCA	ACTGAAATGGATGCATCGTACC
β-actin	TGGATCAGCAAGCAGGAGTA	TCGGCCACATTGTGAACTTT

### 2.6 Western blot

Equal proteins from the cells separated by RIPA reagent were run on 10% SDS-PAGE. After blocking, the membranes were incubated with primary antibodies against adiponectin (ab75989, 1:1,000; Abcam), GLUT-4 (ab33780, 1:1,000; Abcam), IRS-1 (ab46800, 1:10,000; Abcam), phospho-P65 (p-P65, ab28856, 1:1,000; Abcam), P65 (ab32536, 1:1,000; Abcam), phospho-ERK (p-ERK, ab201015; Abcam), ERK (ab184699, 1:10,000; Abcam), phospho-P38 (p-P38, ab178867, 1:1,000), P38 (ab170099, 1:5,000; Abcam), phospho-JNK (p-JNK, ab124956, 1:1,000; Abcam), JNK (ab179461, 1:20,000; Abcam), and β-actin (ab8227, 1:1,000; Abcam), and later with the secondary antibody. After visualization, the protein bands were quantified using Image J software.

### 2.7 Statistical analysis

GraphPad Prism 8.0 software was employed to conduct statistical analysis. The results of each experiment were presented as the mean ± SD of three replicates of each analysis. Analyses of variance were carried out across multiple groups. The significance level was defined as *p* < 0.05.

## 3 Results

### 3.1 Cimifugin inhibits the adipogenesis of 3T3-L1 cells

**Figure 1:**
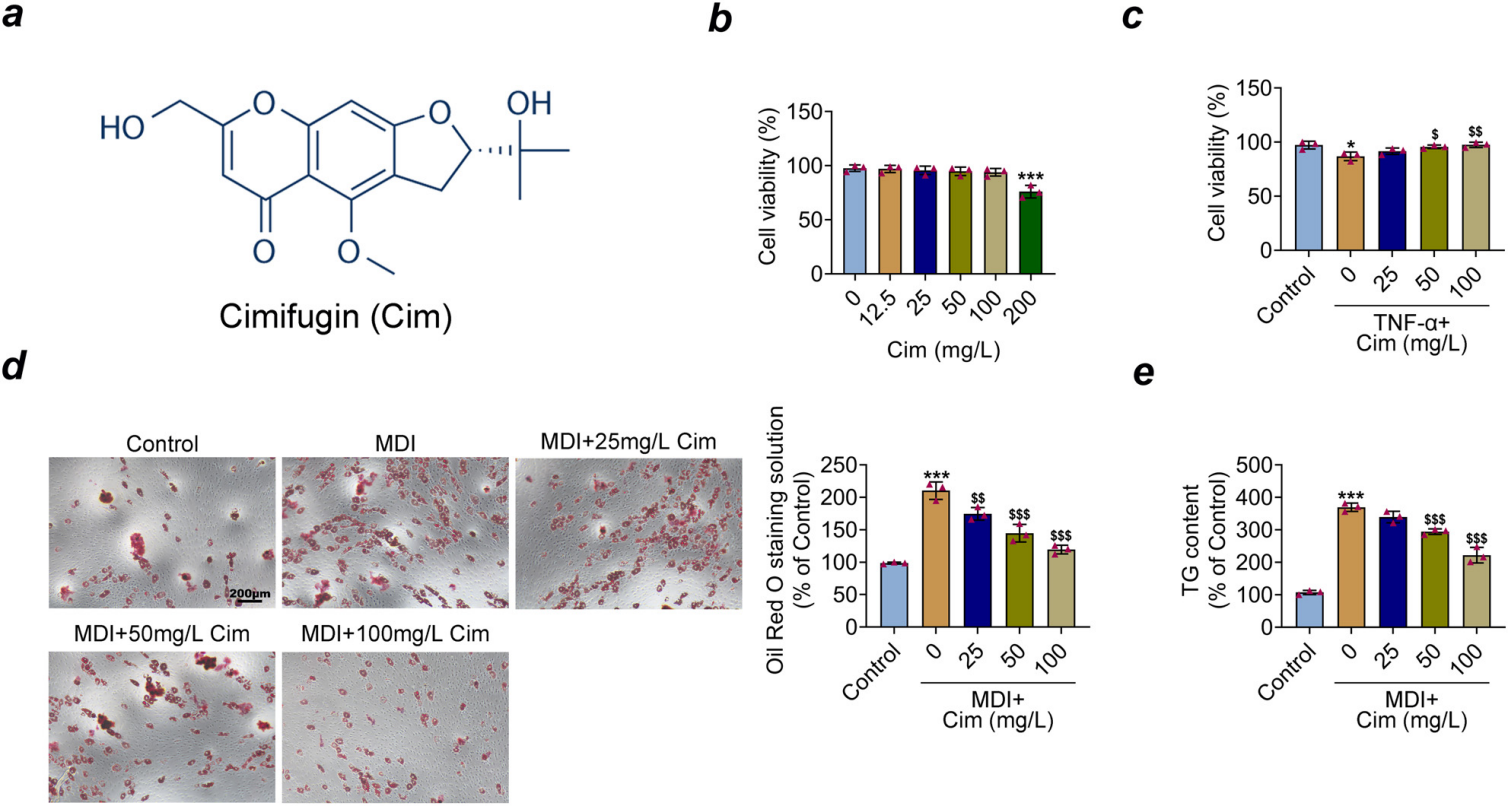
Cimifugin promotes the viability and inhibits the adipogenesis of 3T3-L1 adipocytes exposed to TNF-α. (a) Chemical formula of cimifugin. (b) Cell viability was detected by CCK8 assay in 3T3-L1 adipocytes with different cimifugin concentrations (12.5, 25, 50, 100, and 200 mg/L) in the absence of TNF-α. (c) Cell viability was detected by CCK8 assay in 3T3-L1 adipocytes with different cimifugin concentrations (25, 50, and 100 mg/L) in the presence of TNF-α. (d) A treatment with MDI medium induced the differentiation of 3T3-L1 preadipocytes, while incubating cells with various concentrations (25, 50, and 100 mg/L) of CIM. Then, the formation of lipid droplets was observed by light microscopy after 3T3-L1 adipocytes stained with Oil Red O. (e) Intracellular TG content of 3T3-L1 adipocytes was determined. *p < 0.05, ***p < 0.001, compared with control group; ^$^ *p* < 0.05, ^$$^ *p* < 0.01, ^$$$^ *p* < 0.001, compared with MDI+ group.

The chemical formula of cimifugin is shown in Figure 1a. To explore the role of cimifugin, we first examined cimifugin’s potential toxicity on 3T3-L1 adipocytes with CCK8 assays. As shown in Figure 1b, without TNF-α, concentrations of 0–100 mg/L cimifugin had no obvious effect on 3T3-L1 adipocytes’ viability. However, cimifugin exhibited cytotoxicity by inhibiting 3T3-L1 adipocytes’ activity at concentrations up to 200 mg/L. Therefore, 3T3-L1 adipocytes were treated with three concentrations 25, 50, and 100 mg/L cimifugin in subsequent experiments.

A CCK8 assay was conducted with TNF-α-induced 3T3-L1 adipocytes, and the result showed that TNF-α evidently suppressed cell viability, while cimifugin (50 and 100 mg/L) treatment increased cell viability (Figure 1c). Further, Oil Red O staining of 3T3-L1 cells showed the accumulation of lipid droplets inside the cells after MDI stimulation, which was reduced by cimifugin (25, 50, and 100 mg/L) treatment (Figure 1d). Furthermore, the intracellular TG contents of 3T3-L1 cells were elevated when supplemented with MDI. However, 50 and 100 mg/L cimifugin treatment reduced the TG contents (Figure 1e). Taken together, these data suggest that cimifugin could promote cell viability in TNF-α-induced 3T3-L1 adipocytes and inhibit adipogenesis.

### 3.2 Cimifugin decreases 3T3-L1 adipocytes IR induced by TNF-α

**Figure 2:**
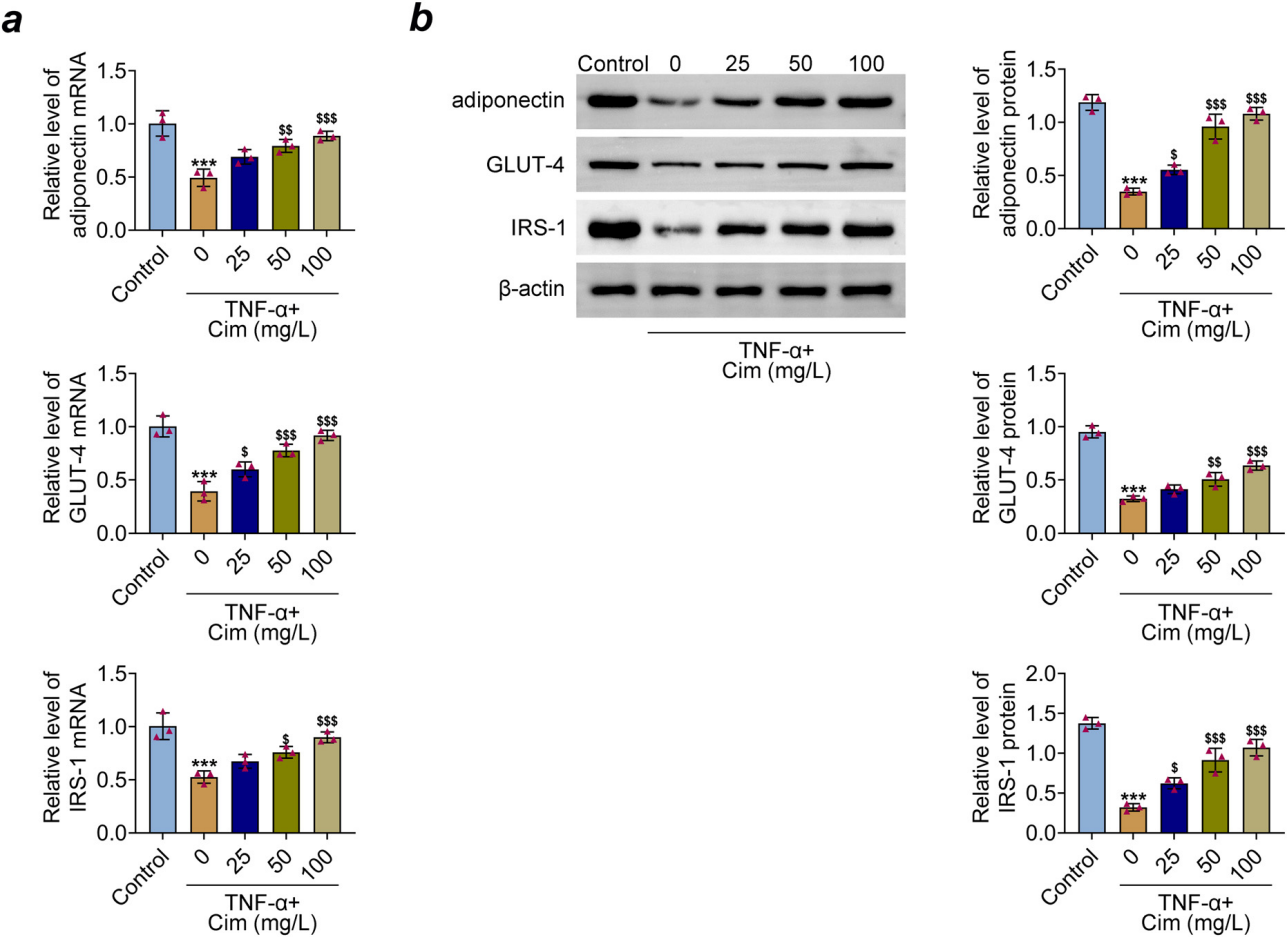
Cimifugin decreases 3T3-L1 adipocytes insulin-resistant induced by TNF-α. (a) mRNA expression of adiponectin, GLUT-4, and IRS-1 was measured by qRT-PCR. (b) Western blot was performed to detect adiponectin, GLUT-4, and IRS-1 protein expression. ***p < 0.001, compared with control group; ^$^ *p* < 0.05, ^$$^ *p* < 0.01, ^$$$^ *p* < 0.001, compared with TNF-α group.

Further, the effects of cimifugin on the IR of 3T3-L1 adipocytes was assessed by detecting the insulin signaling genes, such as adiponectin, GLUT-4, and IRS-1 with qRT-PCR and western blot analysis. As shown in Figure 2a–b, decrease in adiponectin, GLUT-4, and IRS-1 mRNA and protein expression in TNF-α-treated 3T3-L1 adipocytes were observed, but cimifugin pre-treatment ameliorated these alterations. These data suggest that cimifugin attenuated TNF-α-induced IR of 3T3-L adipocytes by improving insulin signaling impairment.

### 3.3 Cimifugin reduces TNF-α-induced inflammation in 3T3-L1 adipocytes

**Figure 3:**
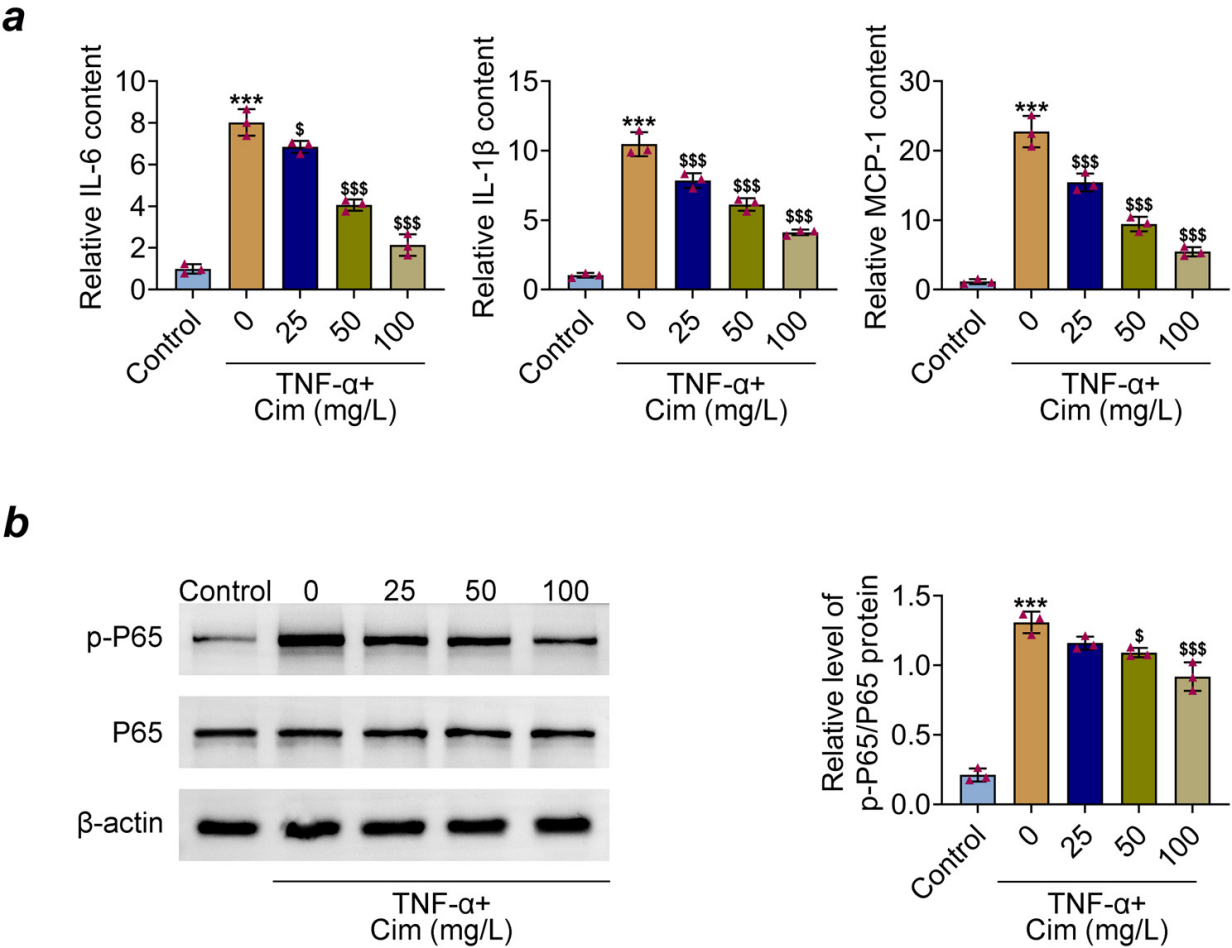
Cimifugin reduces TNF-α-induced inflammation in 3T3-L1 adipocytes. (a) IL-6, IL-1β, and MCP-1 levels were measured by ELISA. (b) Expressions of P65 and p-P65 were determined by western blot. ***p < 0.001, compared with control group; ^$^ *p* < 0.05, ^$$$^ *p* < 0.001, compared with TNF-α group.

The results of ELISA showed that the contents of proinflammatory factors IL-6, IL-1β, and MCP-1 were significantly increased after TNF-α treatment, which were reduced by cimifugin pre-treatment (Figure 3a). Studies have indicated that NF-kB P65 signaling is involved in the response triggered by TNF-α [15,16]. Herein, the effect of cimifugin on P65 phosphorylation level was further studied. As shown in Figure 3b, cimifugin (50 and 100 mg/L) obviously inhibited the elevated expression of p-P65 induced by TNF-α. These data demonstrate that cimifugin probably suppressed the TNF-α-mediated inflammation in 3T3-L1 adipocytes by inactivating P65 pathway.

### 3.4 Cimifugin inhibited the MAPKs pathway

**Figure 4:**
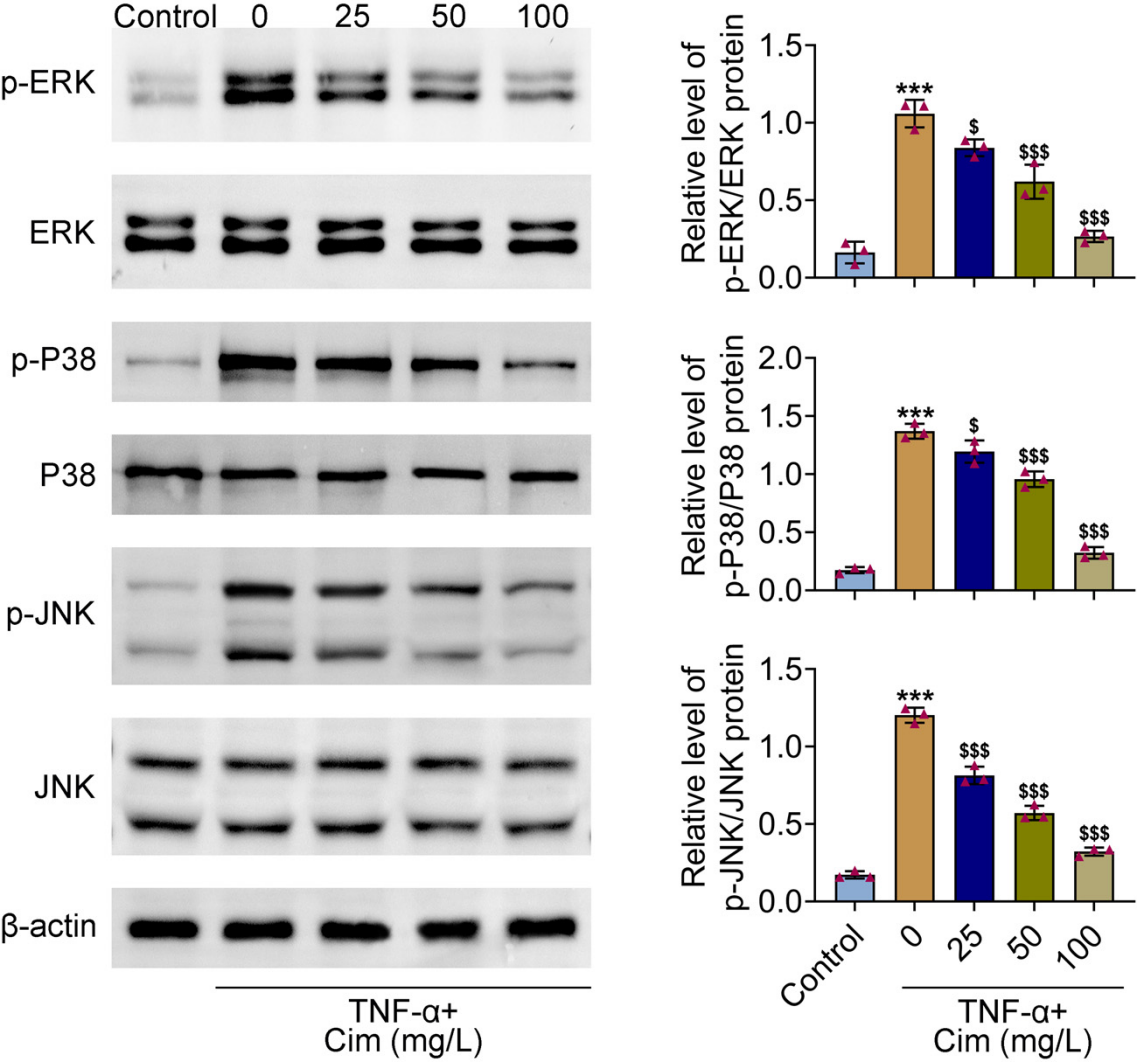
Cimifugin inhibited the MAPKs pathway. Representative blots (left) and quantitative results (right) regarding the protein levels of p-ERK, ERK, p-P38, P38, p-JNK, and JNK detected by western blot. ***p < 0.001, compared with control group; ^$^ *p* < 0.05, ^$$$^ *p* < 0.001, compared with TNF-α group.

Previous studies illustrated that MAPKs activation play a pivotal role in TNF-α-induced adipogenesis and inflammation [17–19]. To explore whether cimifugin had an influence on MAPKs pathway, the phosphorylation levels of ERK, P38, and JNK were assayed by western blot. As a results, TNF-α notably increased the levels of ERK, P38, and JNK phosphorylation. However, pretreatment with cimifugin obviously reduced these increases (Figure 4). These data suggest that cimifugin may exert its biological function through regulating the MAPKs pathway.

## 4 Discussion

The data of this study, for the first time, confirmed that cimifugin could suppress adipogenesis in 3T3-L1 cells. Furthermore, cimifugin attenuated IR and inflammation of 3T3-L1 adipocytes induced by TNF-α. The results also showed that cimifugin inhibited MAPK pathway activation. Thus, these data suggest that cimifugin could reduce obesity-related inflammation and ameliorate obesity-related IR.

IR is the most common metabolic dysfunction related to obesity [20]. The development of IR caused by obesity is connected to the hormones and cytokines produced by adipocytes, including the rise of free fatty acids, TNF, leptin, resistin, and the inadequacy of adiponectin [5,21]. Research has demonstrated that adipocytes’ secretion of TNF-α can prompt the phosphorylation of IRS-1 serine and reduce GLUT-4 expression, resulting in disruptions in insulin-regulated glucose metabolism and the onset of obesity-related IR [22–24]. Therefore, TNF-α is often used to establish IR adipocyte models [17,25,26]. In the present study, 3T3-L1 preadipocytes were induced by MDI to differentiate into adipocytes, and the results found that cimifugin suppressed accumulation of lipids in 3T3-L1 cells. Next, 3T3-L1 adipocytes were treated with TNF-α to induce IR, and the results of western blot indicated that cimifugin could partly reverse the decreased expression of adiponectin, GLUT-4, and IRS-1 TNF-α-treated 3T3-L1 adipocytes.

Studies have suggested that higher levels of inflammatory cell infiltration are present in obese patients, such as TNF-α, IL-6, and MCP-1 [27,28]. These cytokines not only stimulated local inflammation of fat tissues but also caused IR by perturbing the insulin signal transduction pathways [29,30]. A study has reported that cimifugin inhibits LPS-induced release of inflammatory factors and MAPK/NF-kB pathways activation in RAW264.7 cells [31]. Another study confirmed that cimifugin reduced the production of inflammatory factors through inhibiting the NF-kB/MAPK pathway relieving psoriasis [9]. Similarly, our data indicated that cimifugin decreased the levels of pro-inflammatory factors and suppressed P65 phosphorylation expression in TNF-α-stimulated 3T3-L1 adipocytes. Moreover, we also found that cimifugin inhibited TNF-α-caused MAPK pathway activation in 3T3-L1 adipocytes, which reflected in inhibiting ERK, P38, and JNK phosphorylation.

In conclusion, the present study substantiated that cimifugin has the bioactivity of inhibiting adipogenesis and improving TNF-α-induced IR and inflammation in 3T3-L1 adipocytes, and the inhibition of NF-kB/MAPK pathways may possibly explain these functions. These results provided evidence for cimifugin as a medication for preventing and treating obesity and obesity-related IR.
